# Development and use of a Porcine Liver Model (PLM) for robotic liver surgery training

**DOI:** 10.1007/s11701-026-03533-z

**Published:** 2026-06-01

**Authors:** Edoardo Poletto, Laura Alaimo, Mario De Bellis, Bernardo Dalla Valle, Diletta Roman, Tommaso Campagnaro, Simone Conci, Andrea Ruzzenente

**Affiliations:** Department of Surgery, Dentistry, Gynecology and Pediatrics, Division of General and Hepato-Biliary Surgery, University of Verona, Verona, Italy

**Keywords:** Robotic surgery, Liver surgery, Learning curve, Animal model, Ex vivo model

## Abstract

**Objectives:**

Ex-vivo models may be useful in shortening the learning curve and increasing proficiency in robotic surgery. Our team developed an ex-vivo Porcine Liver Model (PLM) for Hepato-Pancreato-Biliary (HPB) robotic surgery training we aimed to validate it.

**Design:**

The model was used in a robotic HPB surgery course. A Likert scale survey was administered to participants, investigating reliability, face validity, content validity and operator confidence.

**Results:**

25 surgeons responded to the survey: 11 residents (median training 4 years), 14 consultants (median practice 5 years). The PLM was used on a step-by-step chain of exercises including cholecystectomy, hepatic hilum dissection, liver mobilization, transection and hepato-jejunal anastomosis. The model was costless and easy to prepare. The average results on the survey were above 4 for reliability, face validity and content validity (4.3, 4.2, 4.5 respectively) and 3.9 for operator confidence; The model scored better among residents, in particular in terms of reliability (4.5 vs 4.1, p=0.05) and face validity (4.4 vs 3.9, p=0.03).

**Conclusions:**

We developed an ex-vivo liver model that may be a valuable tool for robotic surgery training in HPB. This is particularly useful for surgeons with no prior experience with the Da Vinci platform or with minimally invasive liver surgery.

## Background

Minimally invasive liver surgery (MILS) has been gaining consent over the last 25 years, given the very well-known advantages in terms of reduced blood losses, peri- and post-operative complications and shorter length of hospital stay [1]. While this is surely true for laparoscopic liver surgery compared to open surgery, the advantages and applications for the robotic approach are still object of debate, and the recent Robot4HPB consensus guidelines state how the most adequate approach must be chosen considering accessibility, cost-effectiveness, patient-specific factors but also training and expertise [2]. While it has been proven that the learning curve in robotic surgery may be equal to shorter than in laparoscopy, especially due to the possibility for the trainee to be guided and helped thanks to presence of two consoles in most operatory theaters [3, 4], the use of animal models, in vivo or ex vivo, may be helpful, enabling the trainees to become familiar with robotic instruments, understand the effects of manipulation on the tissues and practice using energy devices. Ex vivo models are more commonly used given that they are less expensive and more reproductible, even if less realistic, than in vivo models. Several studies have reported using different animal models for laparoscopic liver surgery training: in particular, goat livers have proven to be highly valuable, due to many similarities between goat and human livers, especially in terms of organ morphology, blood inflow and outflow structures [5, 6]. Porcine models have been used with success in other settings, but porcine liver has not been widely used given that its anatomy presents some differences from that of human’s liver, limiting the types of exercises that can be performed during the training [7, 8], even if it gained consent as a model for liver regeneration models in light of the absence of bile ducts and vessels crossing between left and right hemi-livers [9]. However, the porcine liver is more easily accessible compared to that of other animals, and with adequate preparation it could become a useful tool for robotic HPB surgery training. The present study aims to illustrate the development, use and validation of a Porcine Liver Model (PLM) for robotic hepato-biliary surgical training.

## Methods

### Anatomy of the porcine liver

**Fig. 1:**
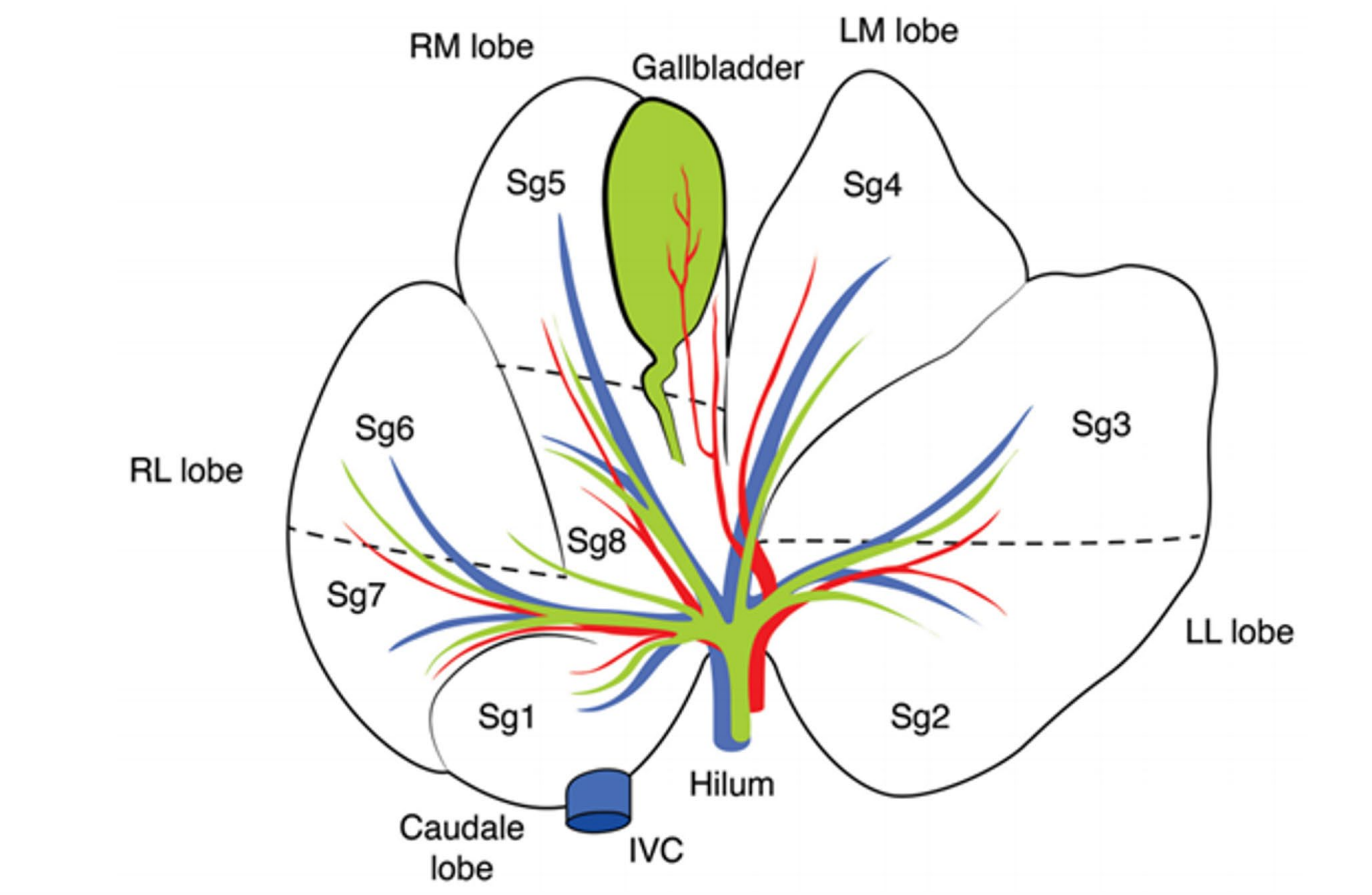
Anatomy of the porcine liver, adapted from Cinelli et al. [9]

The anatomy of the porcine liver shares some similarities and differences with that of the human liver. The porcine liver is leaf shaped, with 5 lobes that roughly resemble the human liver’s sectors: right lateral, right median, left median, left lateral and the caudate lobe, with an identical correspondence of segments’ number in the lobes, as seen in Fig. 1. Vascular inflow is similar, with a similar portal vein and arterial system, that both divide in left and right branches at the hilum; as in humans, no vascular or biliary branches cross between the right and left hemi-livers [9–11]. Unlike the human liver, venous drainage in swine occurs via four main hepatic veins, which run all intrahepatic, with no extrahepatic portion. The inferior vena cava passes through the liver parenchyma and drains directly segment I [12].

## Model preparation

**Fig. 2:**
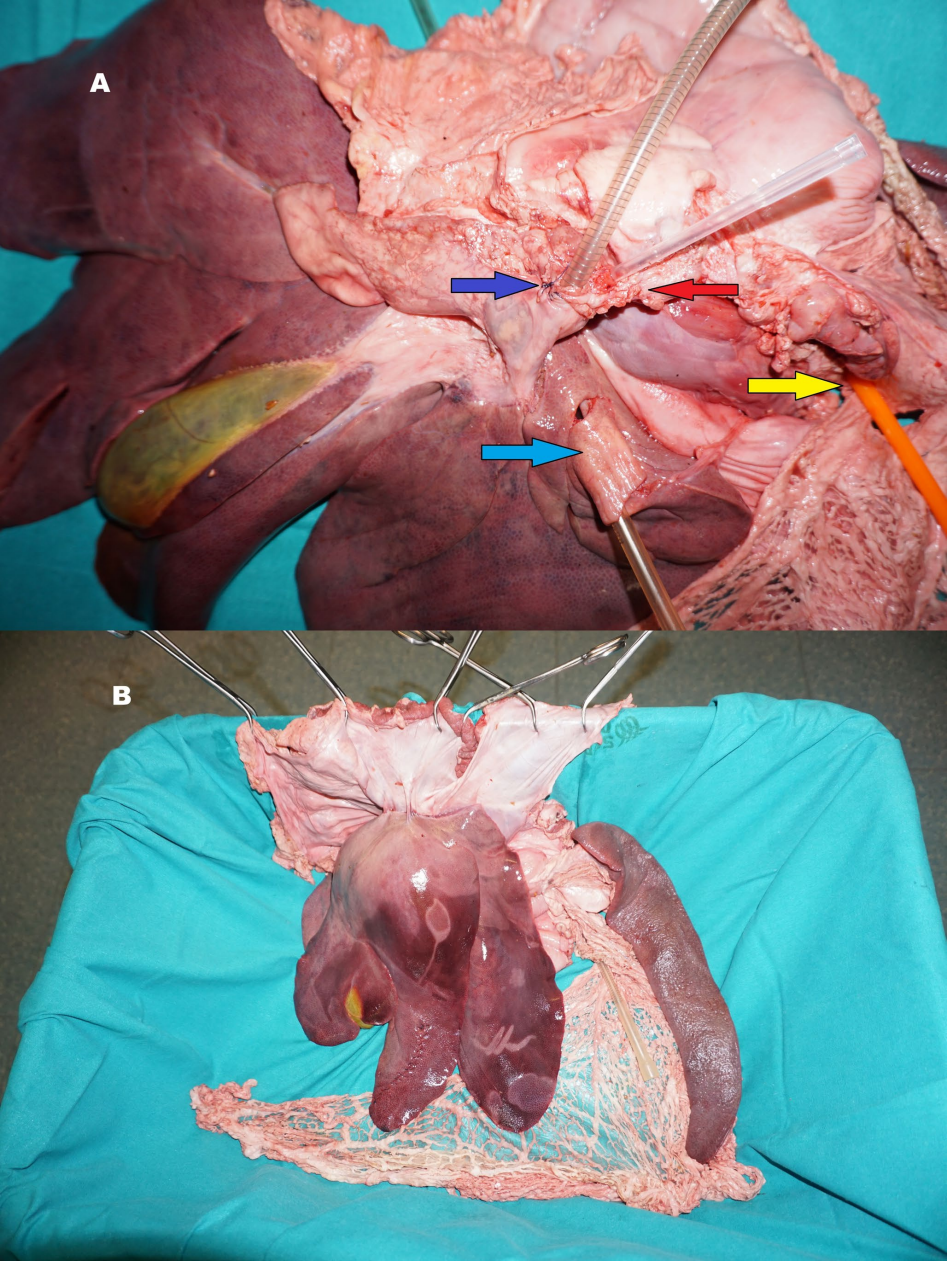
PLM preparation, (**A**) vascular anatomy, showing the cannulation of the portal vein (blue arrow) for perfusion, the hepatic artery (red arrow), inferior vena cava and aorta (light blue and yellow arrows respectively). (**B**) the model is fixed in a box, hung by the diaphragm

The model was first prepared by isolating the infra-hepatic and supra-hepatic segments of the inferior vena cava, which were then ligated using a running suture. Secondly, the common hepatic artery and the portal vein were isolated at the hilum, and the portal vein was cannulated with a perfusion cannula (commonly used in cardiac surgery) and connected to a saline solution colored red, as shown in Fig. 2. Although irrigating the liver with a red-colored solution is not the same as having a continuously perfused model, it enables the proper functioning of monopolar and bipolar instruments and helps maintain the tissue’s realistic texture, preventing it from drying out quickly in the absence of perfusion. Additionally, the red solution enhances more realistic visual feedback for the trainees, especially in case of vascular injury during vessel isolation or transection exercises.

Finally, the model was fixed in a plastic box by the diaphragm using Backhaus forceps, simulating its position in the abdominal cavity inflated by the pneumoperitoneum. The stomach and first duodenal portion, the lesser and greater omentum and the spleen are preserved, both to apply the electric platter for the monopolar instruments, and to allow exercises involving the hilum and hepato-duodenal ligament.

## Model utilization

**Fig. 3:**
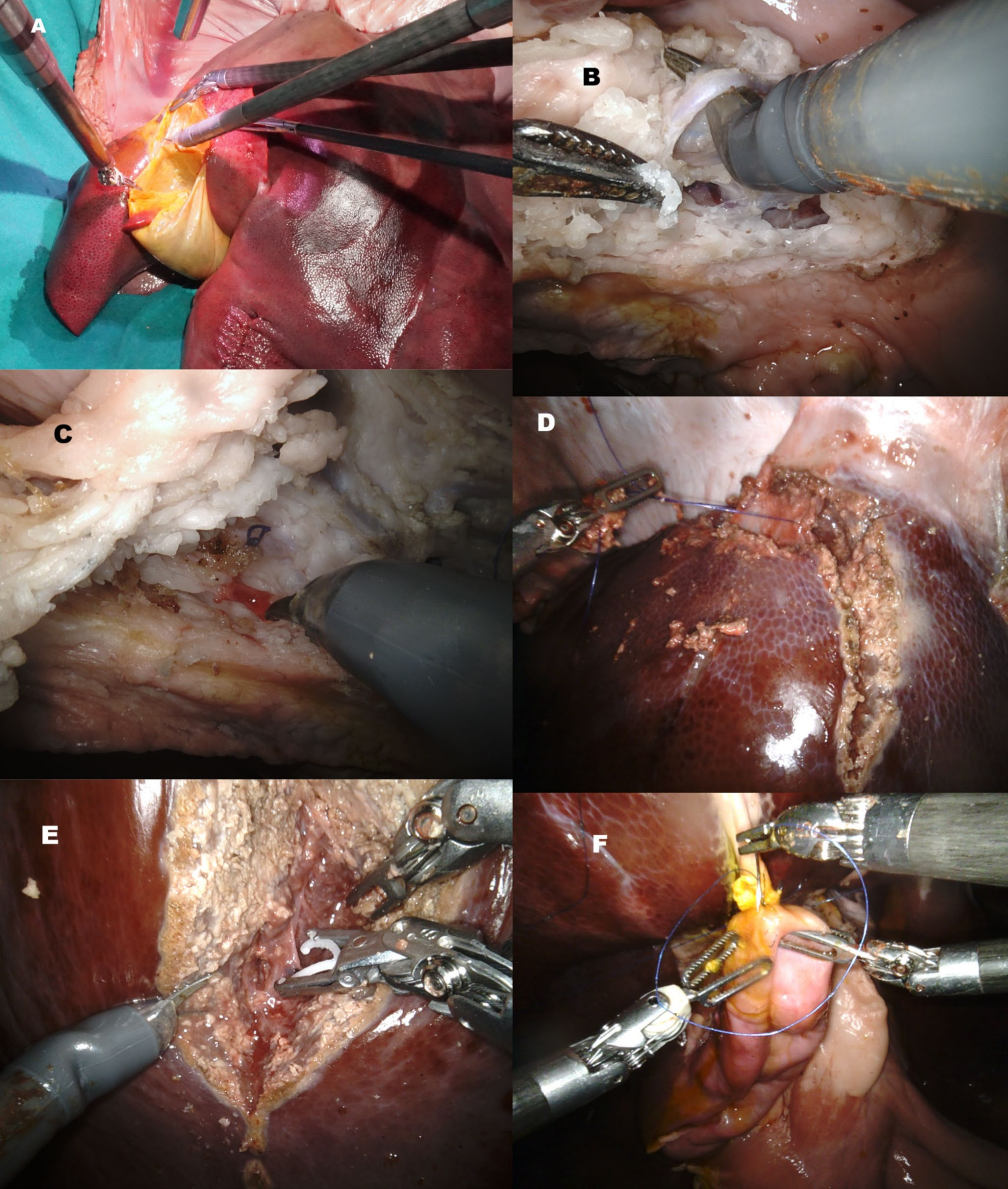
Step by step program of exercises on the PLM: (**A**) cholecystectomy. (**B**) Hepatic hilum dissection with isolation of the hepatic artery. (**C**) Small vascular damage with evidence of “bleeding” thanks to the perfusion of the model. (**D**) Hepatic vein isolation during liver mobilization from the diaphragm. (**E**) Liver transection. (**F**) Hepatic-jejunostomy

During the hands-on part of the three sessions of the course, the participants were divided into pairs, ensuring that everyone had the opportunity to practice various exercises at the console while also staying at the table. We developed a structured training program with a series of 5 step-by-step exercises, depicted in Fig. 3:

1. Cholecystectomy, which was relatively straightforward due to the long cystic duct, a characteristic feature of swine liver anatomy. This exercise allowed the trainees to practice using the scissors and the Maryland forceps, with particular emphasis on utilizing the fourth arm to lift the gallbladder.
2. Hepatic hilum dissection, and thanks to the perfusion of the portal vein with a red-colored solution, a more realistic visualization of vessels and possible damage was provided.
3. Liver mobilization, in which the suspension of the PLM via the diaphragm provided adequate tension, enabling the trainees to perform exercises of liver mobilization and isolation of hepatic veins, even if the veins have no extrahepatic sections.
4. Hepatic transection, with the participants practicing various liver transection techniques, such as using the Maryland and bipolar forceps for the liver parenchyma, as well as hem-o-locks, sutures and a robotic stapler for the main vascular branches.
5. Hepato-jejunal anastomosis. This last exercise could be conducted by suturing the cystic duct, which is wide in diameter and simulates well a non-dilated bile duct, or the isolated main bile duct to the first part of the duodenum, that was preserved in the model.

## Model validation

The model was validated through a survey administered to the participants of a 2-day theoretical and practical course on robotic liver surgery held in five sessions in the years 2022–2025, which included hands-on training with the model. A Likert scale was used in the survey, evaluating 4 criteria (reliability, face validity, content validity and operator confidence, as reported in Table 1) on a scale from 1 (scarce performance of the model) to 5 (excellent performance of the model). Likert scale results have been expressed as mean scores and compared with the independent sample t-test. Statistical analysis was conducted using SPSS.

**Table 1:** Validation questionnaire for PLM with results in the study population expressed as mean scores

Criterion	Question	Total (*n* = 25)
**Reliability**	How reliable do you think the PLM is for robotic liver surgery training?	4.3
**Face Validity**	How realistic do you think the PLM is for robotic liver surgery training?	4.2
**Content validity**	How appropriate do you think the PLM is as a training modality?	4.5
**Operator confidence**	After using this model, do you feel more confident in approaching robotic liver surgery?	3.9

## Results

**Fig. 4:**
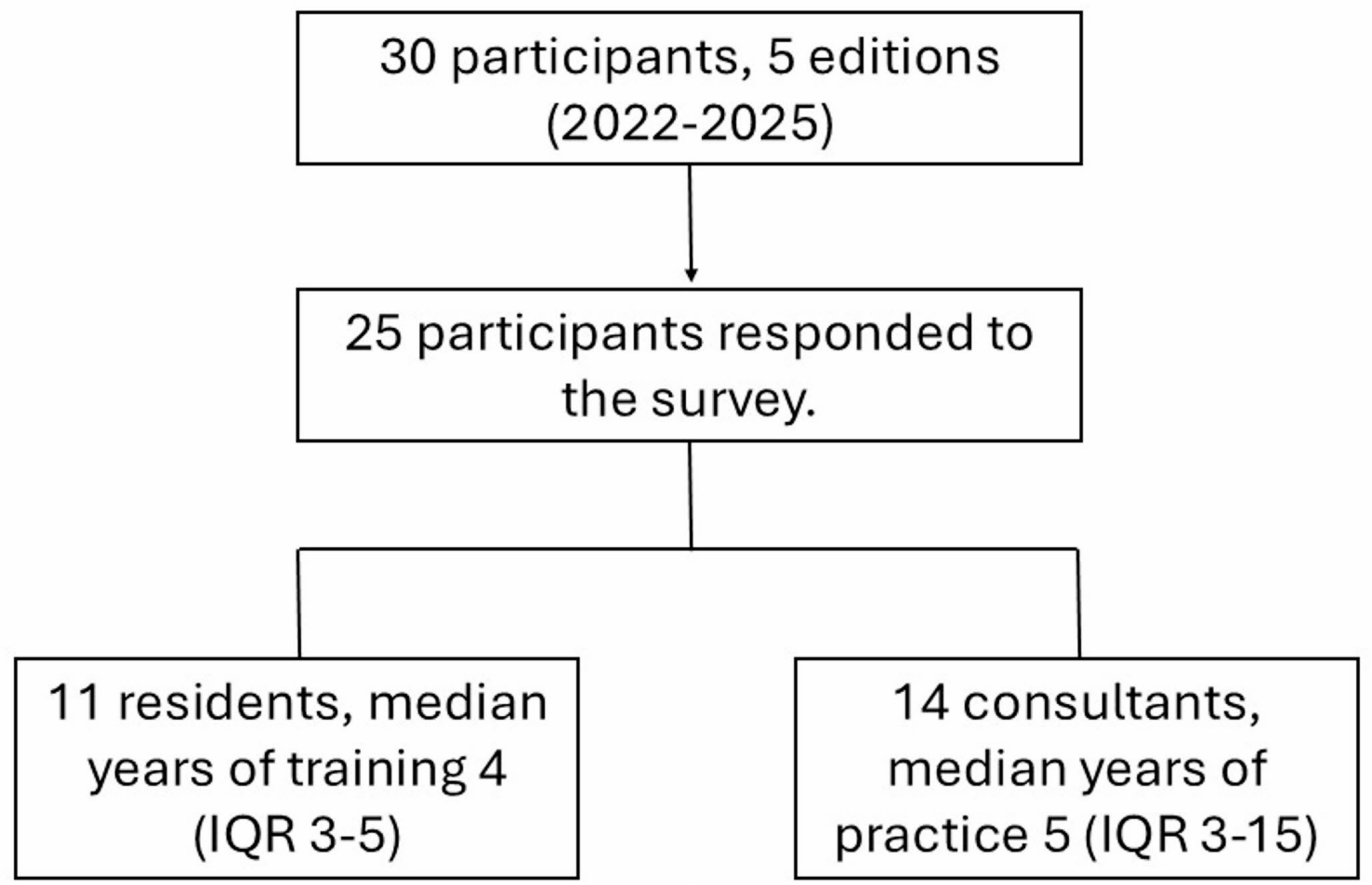
Diagram showing the composition of the study population for the validation of the model

In the study period considered, 30 surgeons participated in the course utilizing the PLM for hepatobiliary robotic training. As reported in Fig. 4 and 25 surgeons responded to the survey regarding the PLM: among them, 11 were residents practicing at a hepatobiliary facility, who have been in their training program for a mean period of 4 years (inter-quartile range 3–5). The remaining 15 participants were consultant surgeons with a wide range of experience, having been appointed for a median of 5 years (inter-quartile range 3–15). A total of 9 (36%) of participants reported no prior experience with the DaVinci^®^ Robotic platform, while 11 participants (44%) reported no experience in HPB surgery with the DaVinci^®^ platform prior to the course.

### Model validation

Mean scores were calculated for every question in the survey, and a comparison was made between residents and consultant surgeons (Table 2):

**Table 2:** Validation questionnaire for PLM, mean scores are presented for residents and consultant surgeons

Criterion	Total (*n* = 25)	Residents (*n* = 11)	Consultants (*n* = 15)	*P* Value
**Reliability**	4.3	4.5	4.1	0.05
**Face validity**	4.2	4.4	3.9	0.03
**Content validity**	4.5	4.7	4.2	0.09
**Operator confidence**	3.9	4.1	3.7	0.20

Means among groups are compared by an independent sample t-test

- Reliability: the question was formulated as follows, “How reliable do you think the PLM is for robotic liver surgery training?”. The performance of the model was very good with a mean score of 4.3. PLM scored higher in reliability among residents, and the difference was almost significant (*p* = 0.05).
- Face Validity: This question enquires how realistic the model felt (“How realistic do you think the PLM is for robotic liver surgery training?”). Performance was very good (mean score 4.2), but in this case the PLM scored significantly higher in the residents’ group than in the consultant group (4.4 vs. 3.9, *p* = 0.03).
- Content Validity: the question read as follows, “How appropriate do you think the PLM is as a training modality?”. Mean score was very good at 4.5, with the residents group giving higher, but not significantly different scores than consultant (4.7 vs. 4.2, *p* = 0.09).
- Operator Confidence: this aspect investigates the impact of the use of the model on the confidence of the surgeon (“After using this model, do you feel more confident in approaching robotic liver surgery?”). Performance was good, the only case in which mean score was below 4 (3.9, with no difference between residents and consultants (4.1 vs. 3.7, *p* = 0.20.

## Discussion

The learning curve for robotic liver surgery demands complete mastery of the robotic system that is used and proficiency with the associated instruments and energy devices [13, 14]. Before performing hepatectomies on live patients, an HPB surgeon should develop a strong familiarity with the robotic platform, therefore, implementing a well-structured training program is essential before undertaking robotic liver surgery, and many advocated how animal models may help in overcoming the learning curve faster in surgery. Although numerous in vivo and ex vivo laparoscopic liver surgery training models have been reported in recent years, there is a lack of published robotic liver surgery training programs [15–17]. Our study aimed to present the development, implementation and validation of an ex vivo Porcine Liver Model (PLM) for robotic hepato-biliary surgical training.

We achieved promising results and received positive feedback, particularly from young and less experienced surgeons who were using the DaVinci^®^ Robotic platform for the first time, and the model showed several advantages.

First of all, our PLM has proven to be cost-effective, very easy to source, and simple to prepare. For the training sessions, we obtained porcine livers from by-products of routine animal slaughter, ensuring that no animals were explicitly sacrificed for the creation of the model. This process incurs no additional costs, as the splanchnic blocks were provided for free, and the only cost was for the retrieval and transport. Clearly this depends mostly on the fact that in our country pig slaughterhouses are widely diffused and easily accessible, while the PLM may not be applicable in geographical areas, lacking such proximity as procuring and transporting the livers would become more challenging and expensive. Unfortunately, this model may also not be suitable for communities with religious objections to pigs.

Moreover, the preparation of the model requires only basic materials such as plastic boxes, cardiac surgery cannulas, any available type of sutures and the red-colored saline solution; even expired cannulas and sutures as well as dismissed surgical instruments can be used for the setup and utilization of the model, further reducing the overall cost of the program.

Additionally, the cost-effectiveness and ease of preparation of our model allows a high degree of repeatability, together with the possibility to perform a series of exercises that are standardizable, given that every participant works on a new model.

Another advantage of our PLM is the size of the livers, which closely resembles that of the human, whereas goat liver is much smaller. Notably, the average volume ranges from 652 to 1120 cm3 compared to 1862 cm3 of human liver, and the weight is about 1810 g versus 1613 g for humans [10, 18]; this gives to the user of the PLM a more “lifelike” experience and helps understanding the proper proportions between the instruments and the structures encountered during liver surgery. However, as previously mentioned, pig liver anatomy differs significantly from that of the human liver, while that of the goat liver is nearly identical. This prompted us to avoid using the PLM to perform entire surgeries, as we believed learning the single steps may reduce the problems connected to anatomical differences; we think this unavoidable defect of our model may be the reason why the PLM scored less in operator confidence than in the other aspects of the survey scores.

The Likert scale used in the survey shows how mean scores are usually higher for residents than consultants: this may be because usually residents have few to no experiences with the robotic platform, let alone with robotic HPB surgery. This is probably particularly true given that when talking about how reliable and realistic (reliability and face validity) the model felt: more experienced surgeons have a better grasp of how a real human liver behaves and feels, therefore recognizing the limitations of the model faster. However, the model seems to perform good in giving confidence to both residents and consultants, and its value in training seems to be appreciated since the higher means scores were for content validity that measures PLM appropriateness for training. So, we can conclude that having a stepwise program administered on a model that permitted both to gain confidence with the platform and learn basic steps applicable to most type of liver surgeries may be more important for residents and younger surgeons than experienced surgeons that may already have confidence with the DaVinci and are just approaching or perfecting HPB robotic surgery.

Similar experiences using ex vivo swine liver models were reported in literature, even if this is, to the best of our knowledge, the first model developed specifically for robotic training. In their experience Liu et al. created a swine liver model that is continuously perfused and used it to train surgeons in laparoscopic suture skills, demonstrating an advantage give by perfusion over dry box training models, and among other things they also demonstrated better results in novices than in experts [19]. Moreover, while we focused only on robotic liver surgery, swine liver models demonstrated their utility also in different type of procedures, such as in the experience reported by Sanada et al. in terms of Split Liver transplantation simulation using a porcine liver model, especially in understanding the biliary anatomy variations that may increase the difficulty of this type of surgery [20].

Certain limitations of the model have been highlighted during its use.Given the already mentioned anatomical differences between human liver and pig liver, as well as the ex-vivo nature of the model, the main limitation was the authenticity of the model. The four separated and thinner lobes, along with the heavy weight of the swine liver, made manipulation more challenging, forcing trainees to perform different handling techniques and more difficult tractions on the liver.

Although the overall cost of setting up the model was not high, another limitation of our PLM is the availability of the Da Vinci Robotic platform. For the courses held in our center, an agreement was reached to bring a robotic platform, a console and all the robotic instruments reserved for animal model training to the training facility for a month, allowing multiple courses robotic general, urological and gynecological surgery to be organized, but this may not be possible in all centers, and the use of animal models requires platforms that are specifically reserved for that scope. In terms of this study, some limitations must be reported. Even though the Likert scale was not developed specifically for this study, but comes from other similar experiences [7], this tool may result “subjective”, and not a proper instrument for validating this study. Another limitation is the low number of participants, since the experience is still initial and a low number of participants were admitted to each session of the robotic HPB Surgery course.

## Conclusions

In conclusion, we developed an ex-vivo liver model that may be a valuable tool for robotic surgery training in HPB. This is particularly useful for surgeons with no prior experience with the Da Vinci platform or with minimally invasive liver surgery.

## Data Availability

No datasets were generated or analysed during the current study.

## Abbreviations

HPB: Hepato-pancreato-biliary
PLM: Porcine liver model
MILS: Minimally invasive liver surgery
