# Correction: Development and use of a Porcine Liver Model (PLM) for robotic liver surgery training

**DOI:** 10.1007/s11701-026-03593-1

**Published:** 2026-07-20

**Authors:** Edoardo Poletto, Laura Alaimo, Mario De Bellis, Bernardo Dalla Valle, Diletta Roman, Tommaso Campagnaro, Simone Conci, Andrea Ruzzenente

**Affiliations:** Department of Surgery, Dentistry, Gynecology and Pediatrics, Division of General and Hepato-Biliary Surgery, University of Verona, Verona, Italy

**Correction to: Journal of Robotic Surgery (2026) 20:572**

10.1007/s11701-026-03533-z

In the original version of the article, the author names Hepato-Biliary Surgery and Andrea Ruzzenente General were inadvertently added by mistake.

The original article has been corrected.

